# The MUC1 Extracellular Domain Subunit Is Found in Nuclear Speckles and Associates with Spliceosomes

**DOI:** 10.1371/journal.pone.0042712

**Published:** 2012-08-08

**Authors:** Priyadarsina Kumar, Louise Lindberg, Twanda L. Thirkill, Jennifer W. Ji, Lindsay Martsching, Gordon C. Douglas

**Affiliations:** Department of Cell Biology and Human Anatomy, School of Medicine, University of California Davis, Davis, California, United States of America; Northwestern University Feinberg School of Medicine, United States of America

## Abstract

MUC1 is a large transmembrane glycoprotein and oncogene expressed by epithelial cells and overexpressed and underglycosylated in cancer cells. The MUC1 cytoplasmic subunit (MUC1-C) can translocate to the nucleus and regulate gene expression. It is frequently assumed that the MUC1 extracellular subunit (MUC1-N) does not enter the nucleus. Based on an unexpected observation that MUC1 extracellular domain antibody produced an apparently nucleus-associated staining pattern in trophoblasts, we have tested the hypothesis that MUC1-N is expressed inside the nucleus. Three different antibodies were used to identify MUC1-N in normal epithelial cells and tissues as well as in several cancer cell lines. The results of immunofluorescence and confocal microscopy analyses as well as subcellular fractionation, Western blotting, and siRNA/shRNA studies, confirm that MUC1-N is found within nuclei of all cell types examined. More detailed examination of its intranuclear distribution using a proximity ligation assay, subcellular fractionation, and immunoprecipitation suggests that MUC1-N is located in nuclear speckles (interchromatin granule clusters) and closely associates with the spliceosome protein U2AF65. Nuclear localization of MUC1-N was abolished when cells were treated with RNase A and nuclear localization was altered when cells were incubated with the transcription inhibitor 5,6-dichloro-1-*b*-d-ribofuranosylbenzimidazole (DRB). While MUC1-N predominantly associated with speckles, MUC1-C was present in the nuclear matrix, nucleoli, and the nuclear periphery. In some nuclei, confocal microscopic analysis suggest that MUC1-C staining is located close to, but only partially overlaps, MUC1-N in speckles. However, only MUC1-N was found in isolated speckles by Western blotting. Also, MUC1-C and MUC1-N distributed differently during mitosis. These results suggest that MUC1-N translocates to the nucleus where it is expressed in nuclear speckles and that MUC1-N and MUC1-C have dissimilar intranuclear distribution patterns.

## Introduction

The mucin, MUC1, is a large Type 1 transmembrane glycoprotein expressed at the apical surface of epithelial cells and over-expressed (and under-glycosylated) by several epithelial tumor cells [1], [2], [3]. MUC1 is also expressed by some hematopoietic cells [4]. Understanding how MUC1 regulates cell function continues to occupy current research efforts and the importance of MUC1 as an oncogene is highlighted by the fact that it is the subject of vaccine development for treatment of several human cancers. After synthesis, MUC1 undergoes autoproteolytic cleavage at a sea urchin sperm protein, enterokinase, agrin (SEA) domain to form two polypeptides (an N-terminal derived alpha subunit – termed MUC1-N and a C-terminal derived beta subunit – termed MUC1-C) which non-covalently associate [5]. After transit through apical recycling endosomes, the MUC1 heterodimer is inserted in the apical plasma membrane [6] where it has multiple functions. MUC1-N consists of 1000–2000 amino acids arranged as variable numbers of tandem repeats (VNTR) that extend far above the cell surface. Extensive O-linked glycosylation of the extracellular domain is largely responsible for the anti-adhesive and lubricant properties of MUC1 on mucosal surfaces. MUC1-N can be shed from the cell surface following proteolytic cleavage or subunit dissociation [7]. It has been suggested that the MUC1-C subunit (comprising a short extracellular domain that excludes any terminal repeats, a transmembrane domain, and the cytoplasmic domain) can function as a receptor that triggers intracellular signaling [8], [9]. The cytoplasmic domain consists of 72 amino acids and has several potential phosphorylation sites. In cancer cells, polarized MUC1 expression at the apical surface is lost and MUC1-C can interact with key signaling molecules such as EGFR, Wnt–β-catenin, p53, and NF-κB [2], [3].

The antibody CT2 reacts with the cytoplasmic domain of MUC1-C and immunohistochemical studies generally show plasma membrane/cytoplasmic staining [10], [11]. However other studies using CT2 or other MUC1 cytoplasmic domain-specific antibodies demonstrate that MUC1-C can be transported to the nucleus where it is involved in the regulation of transcription [3], [12], [13], [14], [15], [16], and to mitochondria [17]. Transport of MUC1-C to the nucleus is dependent on the CQC motif which is required for MUC1 oligomerization and direct interaction with importin β and nucleoporin-62 (Nup62) [12]. Wei and Kufe showed that p53 immunoprecipitation of nuclear extracts pulled down MUC1-C but not the extracellular domain subunit [18]. Incubation of ZR-75 mammary tumor cells with heregulin induced the translocation of γ-catenin and MUC1-C (but not MUC1-N) to the nucleolus [16]. These results suggest that MUC1-C may belong to an increasing group of plasma membrane proteins that can be translocated to the nucleus after proteolytic cleavage or subunit dissociation at the plasma membrane [19], [20], [21].

Because of its involvement in intracellular signaling and transcriptional regulation, there has been much focus of attention on MUC1-C. There is also a frequent assumption that only MUC1-C, and not MUC1-N, translocates to the nucleus [13], [16], [18], [22]. A large number of immunohistochemical studies have been carried out on normal epithelial and tumor tissues using panels of well-characterized antibodies against the MUC1-N subunit [23]. Most of these antibodies recognize epitopes within the 20-amino acid VNTR region of MUC1-N and antibody reactivity often varies depending on the degree of O-linked glycosylation. While the intensity of staining varies depending on the tissue and antibody used, reactivity is, as expected, generally described as being associated with the apical plasma membrane in most normal tissues [10], [24], [25]. However, intracellular expression of MUC1 extracellular domain antibody reactivity is found in cancer tissue [11], [26] and in normal uterine epithelial cells [27]. Mahanta et al [9] reported that the MUC1 extracellular domain was found predominantly in the cytoplasm in normal fallopian tube epithelium and in the cytoplasm of epithelia from cancerous breast, lung, and colon tissue. The same study showed that the predominant form of MUC1 expressed on the surface of these cells was in fact the low molecular weight beta (MUC1-C) subunit resulting from cleavage and/or shedding of the alpha subunit (MUC1-N) [9]. In a different study, Western blotting analysis of subcellular fractions from breast cancer tissue found MUC1 extracellular domain immunoreactivity in membrane, cytosol, and pellet/nuclear fractions [11]. A recent study concluded that full length MUC1 interacts with the transcription factor p65 and that this complex binds to cytokine promoter regions in the nucleus of breast cancer cells [28]. These observations begin to challenge the idea that only MUC1-C translocates to the nucleus and is involved in gene regulation.

Villous trophoblasts form the epithelium that covers the surface of the placenta and interface directly with maternal blood. We and others have reported the expression of MUC1 by trophoblasts in human and rhesus monkey placental tissue as well as in isolated trophoblasts [29], [30], [31]. Our immunofluorescence microscopy studies using the CT2 cytoplasmic domain-specific antibody showed a weak diffuse staining pattern in isolated rhesus trophoblasts consistent with a cytoplasmic/plasma membrane localization. On the other hand when trophoblasts were stained using the MUC1 extracellular domain-specific antibody B27.29, in addition to some cytoplasmic/membrane staining, a discrete punctate fluorescence pattern was observed, apparently associated with the nucleus [30].

While this unexpected and discordant distribution of CT2 and B27.29 staining in trophoblasts was not investigated further at the time, we have now carried out a detailed analysis of the intracellular distribution of MUC1-N to test the hypothesis that it is expressed in the nucleus. The results obtained using three different well-characterized antibodies against the MUC1 VNTR region along with confocal microscopy, siRNA/shRNA, and subcellular fractionation studies show that the MUC1 extracellular domain is found within the nucleus of trophoblasts and several other normal and cancerous epithelial cells. In addition, we show for the first time that nuclear MUC1-N localizes to nuclear speckles (interchromatin granule clusters) and associates closely with the spliceosome component, U2AF65.

## Materials and Methods

### Antibodies

The mouse monoclonal antibodies B27.29 and DF3 were kindly provided by Fujirebio Diagnostics, Inc., Malvern, PA. B27.29 was originally raised against a partially purified mucin fraction from the ascites fluid of a cancer patient. This antibody recognizes a sequence within the MUC1 tandem repeat domain [32]. Antibody DF3 was raised against a membrane-enriched fraction of a human breast carcinoma metastatic to liver [33] and also recognizes an epitope within the tandem repeat domain. The Armenian hamster monoclonal antibody CT2 [10] recognizing an epitope in the cytoplasmic tail of MUC1 was generously provided by Dr. Sandra Gendler, Mayo Clinic, Arizona, or was purchased from Thermo Fisher, Kalamazoo, MI (product no. HM-1630-P0). The mouse monoclonal antibody HMFG1 (ab707475) recognizing a sequence in the MUC1 tandem repeat domain [6], matrin-3 (ab51081), and U2AF65 (ab37483) were obtained from Abcam (Cambridge MA). Mouse monoclonal antibodies against spliceosomes (MAB1286), cytokeratin 7 (MAB3226), and β1 integrin (MAB1965) were obtained from Millipore (Billerica MA). Antibodies against GAPDH (6C5; sc-32233), Sp1 (sc-59), lamin B1 (sc-20682), and U1snRNP70 (sc-25371) were obtained from Santa Cruz Biotechnology (Santa Cruz CA). Monoclonal antibody against sc35 (556363) was obtained from BD Biosciences (San Jose CA). Additional details relating to the primary antibodies used and their concentrations are shown in Table 1.

**Table 1:** Primary antibodies used in this study.

Name	Type	Specificity	Conc. Western blot	Conc. IFA, cells	Conc. IFA, tissue	Source	Catalog #
HMFG1	M	MUC1 VNTR	0.11 µg/ml	3.26 µg/ml	11.4 µg/ml	Abcam	ab70475
B27.29	M	MUC1 VNTR	1.02 µg/ml	2.04 µg/ml	3.4 µg/ml	Fujirebio	201–485
DF3	M	MUC1 VNTR	1.9 µg/ml	5 µg/ml	NA	Fujirebio	NA
CT2	Armenian hamster Monoclonal	MUC1 cytoplasmic tail	0.04 µg/ml	0.67 µg/ml	0.4 µg/ml	Thermo Fisher	HM-1630-P0
β1-integrin	M	β1-integrin, a.a. 82–87	Approx. 1.75 µg/ml(ascites fluid)	Approx. 35 µg/ml (ascites fluid)	Approx. 35 µg/ml (ascites fluid)	Chemicon	MAB1965
U2AF65	P	epitope corresponding toa.a 1–100 of U2AF65.	0.04 µg/ml	0.4 µg/ml	NA	Abcam	ab37483
Spliceosome	M	Spliceosomes	1/1,000 dilution(ascites fluid)	1/150 dilution(ascites fluid)	NA	Millipore	MAB1286
Sp1	P	epitope corresponding toa.a 528–546	0.1 µg/ml	NA	NA	Santa Cruz Biotechnology	sc-59
Matrin-3	P	Epitope corresponding toa.a 1–50	NA	1 µg/ml	NA	Abcam	ab51081
Cytokeratin7	M	Cytokeratin7	0.5 µg/ml	NA	NA	Millipore	MAB3226
Lamin B1	P	Epitope corresponding toa.a 401–490	NA	1 µg/ml	NA	Santa Cruz	sc20682
SC35	M	SC35	1 µg/ml	NA	NA	BD Bioscience	556363
U1 snRP70	P	C-terminus	0.2 µg/ml	NA	NA	Santa Cruz Biotechnology	sc-9571
GAPDH	M	GAPDH	0.025 µg/ml	NA	NA	Santa Cruz Biotechnology	sc-32233

Abbreviations used in Table 1. M, mouse monoclonal; P, rabbit or goat polyclonal; NA, not applicable; a.a., amino acid; Conc., concentration.

### Cell Culture

Trophoblast cells were isolated from Rhesus monkey (*Macaca mulatta*) placental tissue (Gestation Day: 40–65 days) using procedures we have previously described [34], [35]. All procedures involving animals were performed in accordance with the NIH Guide for the Care and Use of Laboratory Animals and under the approval of the University of California Davis, Animal Care and Use Committee (Animal Protocol #15639). These cells are 95% cytokeratin 7-positive and 5% vimentin-positive. Cells were cultured in a humidified glove box (COY Laboratory Products, Grass Lake MI) at 4% oxygen, 5% CO2) and 37°C in a culture media containing DMEM/F12, 10% serum, 200 mM Glutamine, 1% penicillin/streptomycin.

MCF-7 (a human breast epithelial adenocarcinoma cell line [36]), BeWo and Jar (human trophoblast-derived choriocarcinoma cell lines) cells [37] were obtained from ATCC. COS-7 cells transfected with MUC1 WT (COS7.MUC1) [38] were kindly provided by Dr. Sandra Gendler (Mayo Clinic, Arizona). The full length MUC1 construct used to transfect these cells also contained a Flag tag near the 5′ end [38]. Culture medium for MCF-7 and Jar cells was: DMEM (high glucose), 10% FBS, 1% penicillin/streptomycin, 0.1% gentamicin, 200 mM glutamine, and 1% sodium pyruvate. Culture medium for BeWo cells was: Kaighn’s F-12K, 10% FBS, 200 mM glutamine, 1% penicillin/streptomycin and 0.1% gentamicin. Culture medium for COS7.MUC1 cells was the same as MCF-7 medium except 1 µg/ml puromycin was added. Primary cultures of normal human mammary epithelial cells (240LB, passage 5–6) were kindly provided by Dr Martha Stampfer (Berkeley Laboratory, Lawrence Berkeley National Laboratory, Berkeley, CA). 240LB cells are derived from reduction mammoplasty specimens. Details of the mammary epithelial cell isolation and cell culture conditions can be found at http://hmec.lbl.gov.

### Immunocytochemistry

For immunofluorescence staining, adherent cells on 8-chamber glass LabTek culture slides were fixed with ice-cold 3.7% paraformaldehyde for 5 min and permeabilized using 0.2% Triton X-100. The slides were then blocked in PBS containing 0.2% gelatin and incubated overnight at 4°C with primary antibodies followed by incubation with secondary antibodies for 1 h. Primary antibody controls consisting of isotype-matched mouse, rabbit, or Armenian hamster immunoglobulins (Abcam, Cambridge, MA) were always included. Primary antibodies were detected using FITC-labeled goat anti-hamster IgG (Abcam, Cambridge MA), AlexaFluor-488-labeled, AlexaFluor-594-labeled, or AlexaFluor-647-labeled goat anti-mouse IgG antibodies (Invitrogen Corporation, Carlsbad CA; secondary antibodies were diluted 1∶400). Nuclei were stained using 4′,6-diamidino-2-phenylindole (DAPI). The slides were coverslipped using glycerol-based medium containing anti-fade reagent and viewed using a widefield or confocal microscope. Acquisition settings were kept constant for each antibody and its respective control. Images were pseudocolorized using Adobe Photoshop. The primary antibodies used and their concentrations are shown in Table 1.

Confocal images were captured with an Olympus Fluoview 1000 system using a 60X water objective (N/A = 1.2). The pinhole size and optical section thickness were set automatically for optimal Nyquist sampling. Z-stacks generally consisted of between 15 and 20 images with 0.54 µm increments. Image size was 800×800 pixels, and the pixel width was 98 nm. Image capture conditions for each channel were set for maximum brightness with no pixel saturation.

For colocalization studies, confocal two-color images were captured sequentially using the same exposure times for both channels. To further avoid bleed-through artifacts, AlexaFluor-647 and AlexaFluor-488 conjugated secondary antibodies were used.

### In Situ Proximity Ligation Assay

Cells were cultured for 48 h in eight-chamber glass LabTek slides and fixed with 3.7% formaldehyde with 0.2% Triton X-100. Cells were then incubated with blocking solution consisting of PBS and 0.2% gelatin with 0.5% azide. The fixed cells were incubated overnight at 4°C with antibody B27.29 and anti-U2AF65. The slides were washed three times with PBS for 15 min total with gentle shaking and then incubated with secondary antibodies covalently linked to proximity probes (Olink Bioscience, Uppsala, Sweden) for 2 h at 37°C in a humidified chamber. After washing as above, hybridization, ligation, and rolling-circle amplification were performed using the Duolink detection kit (Olink Bioscience) and following the manufacturer’s instructions. Nuclei were stained using DAPI and slides were mounted with glycerol-based mounting medium. Images were captured using a widefield fluorescence microscope. Several controls were included. For these, reactions were carried out in which (a) isotype-matched immunoglobulins were used in place of primary antibodies, (b) primary antibodies were omitted, or (c) proximity probes were omitted.

### Immunohistochemistry

Immunohistochemical staining was performed on cryosections of rhesus monkey colon. Tissues were immersed in OCT and kept frozen at −80°C. Cryosections (6 µm) were prepared and fixed in 3.7% formaldehyde for 20 minutes. Sections were blocked with 0.6 mg/ml human IgG for 1 hour and then incubated overnight at 4°C with antibody B27.29 or HMFG1. Control sections were incubated with matched non-immune immunoglobulin. After washing, the sections were incubated with appropriate secondary antibodies labeled with AlexaFluor-488, AlexaFluor-594, or AlexaFluor-647 for 30 minutes. DAPI was included to stain nuclei. After further washing, the sections were mounted in glycerol-based medium and viewed with a widefield or confocal fluorescence microscope.

### Transfection with siRNAs

Cells were transfected individually with 25 nM each of On-TARGET PLUS SMART pool MUC1 siRNA (L-004019-01-005, Dharmacon), MUC1 siRNA 599 (SR303004A, Origene, Rockville MD), MUC1 siRNA 600 (SR303004B Origene), MUC1 siRNA 601 (SR303004C, Origene), GAPDH siRNA (L-004253-01-000 Dharmacon) and On-TARGET PLUS non-targeting siRNA pool (D-001810-10-20 Dharmacon) using DharmaFECT siRNA Transfection Reagent 1 (Dharmacon, Thermo Scientific, Waltham MA). The transfection reagent/siRNA was removed after 5 h and fresh medium containing 25 mM HEPES was added. Media were changed every day and cells were harvested 5 days post-transfection. The cells were then lysed and analyzed by Western blotting (see below) and immunocytochemistry as described above. Reagent-only (no siRNA) and medium-only controls were also included.

The regions of MUC1 targeted by the different siRNAs (based on accession number J05581) were as follows: Dharmacon pool: 1 1292 bp-1309; 2 1123–1141 3 1642–1661 4 1053–1071. Origene: 599 921–945; 600 56–87; 601 1337–1362.

### Transfection with shRNA

Lentiviral MUC1shRNA expression plasmid (TRCN0000122938, SHCLNG-NM_002456) and scrambled shRNA plasmid (pLKO.1-puro Non-Mammalian shRNA Control Plasmid DNA SHC002) were purchased from Sigma-Aldrich, St. Louis MO. HEK293T cells were cotransfected with 10 µg Lentiviral plasmids (MUC1 or scrambled), 7.5 µg packaging plasmid and 5 µg envelope plasmid using Lipofectamine 2000 as transfection reagent. Culture media were harvested 48 h post transfection, clarified by centrifugation at 500×g for 10 min and stored as aliquots at −80°C. JAR cells were transduced with virus particles in the presence of 4 µg/ml polybrene for 5 h and then selected with 1 µg/mL puromycin. Selected cells were maintained in culture medium containing 1 µg/mL puromycin.

### Western Blotting

Total lysates were obtained by incubating cells in RIPA lysis buffer (Thermo Scientific) supplemented with 1% Protease Inhibitor Cocktail (Sigma-Aldrich, St. Louis MO) and 1 mM EDTA for 1 h at 4°C. The lysate was centrifuged at 13,000 x g for 10 min at 4°C and a protein assay was performed on the supernatant using the BCA assay kit (Thermo Scientific). The supernatant was mixed with NuPAGE LDS sample buffer (Invitrogen) containing DTT and heated at 70°C for 10 min. The samples were centrifuged at 13,000×g for 2 min and loaded on 3–8% Tris-Acetate SDS-NuPAGE gels (Invitrogen) at 10–30 µg protein per lane. After electrophoresis the proteins were transferred to PVDF (BioRad, Hercules CA). The membrane was blocked for 1 h in 0.5% casein in TBST, (Tris-buffered saline plus 0.5%Tween 20). The blocked membrane was incubated overnight with the primary antibody (concentrations are shown in Table 1) in 0.1% casein in TBST, then washed and incubated with secondary antibody labeled with horseradish peroxidase (Thermo Scientific). After further washing, the membrane was incubated with chemiluminescent substrate (SuperSignal West Dura; Thermo Scientific or WesternBright Quantum, E&K Scientific) and then exposed to a Kodak imager (Kodak Imaging Systems, New Haven CT).

### RT-PCR

Total RNA was obtained from cells using the RNeasy Plus Mini kit (Qiagen, Valencia CA) and cDNA synthesized from 1 µg of RNA using SuperscriptII Reverse transcriptase (Invitrogen, Carlsbad CA) was used in a PCR reaction using primers [39] corresponding to full length MUC1: Forward primer: 5′ TGCATCAGGCTCAGCTTCTA 3′ and Reverse primer: 5′ GAAATGGCACATCACTCACG3′. GAPDH was used as a control and was amplified using: Forward primer: 5′ GCCAGCATCGCCCCACTTGA 3′ and Reverse primer 5′ CGGTCGTAGCGGGGTGAACT 3′. Both were amplified using AccuPower PCR premix (Bioneer, Alameda CA) at an annealing temperature of 60°C for 35 cycles.

### Sub-cellular Fractionation

Subcellular fractionation was carried out using the Subcellular Protein Fractionation Kit (Thermo Scientific, Rockford IL) as described by the manufacturer. The procedure yields (1) a cytosolic fraction, (2) a membrane fraction, (3) a nuclear soluble fraction, (4) a nuclear chromatin bound fraction, and (5) a cytoskeletal fraction. Fraction purity was assessed by Western blotting using antibodies against GAPDH, β1-integrin, Sp1 transcription factor, U2AF65, and spliceosomes. Equal volumes of each fraction were loaded onto the NuPAGE gel and Western blotting was performed as described above.

### Immunoprecipitation

Nuclear extracts of JAR cells were prepared using the sub-cellular fractionation kit (Thermo Scientific, Rockford IL) described above. The extracts were incubated with either anti-MUC1 (DF3) or control mouse IgG1 antibodies overnight at 4°C. The immune complexes were precipitated with ProteinA/G plus agarose (Santa Cruz Biotechnology, CA), washed with wash buffer (50 mM Tris-HCl pH 8.0, 200 mM NaCl plus protease inhibitors) and eluted in 1X LDS sample buffer. Immunoprecipitated proteins were resolved on 3–8% Tris Acetate gels and analyzed by Western blotting as described above.

### Nuclease Digestion

Nuclease digestion was performed according to Spector *et al.* [40]. Cells were cultured until semi-confluent and then fixed and permeabilized using ice-cold methanol for 2 min. The fixed cells were washed using PBS and then incubated with RNase A (100 µg/mL) for 2 h at 25°C. The cells were washed with PBS and processed for immunocytochemistry as described above.

### DRB Treatment

Cells were incubated with 100 µM 5,6-dichloro-1-*b*-d-ribofuranosylbenzimidazole (DRB) for 2 h at 37°C and then processed for immunocytochemistry as described above.

### Nuclear Speckle Isolation

Nuclear speckles (interchromatin granular clusters) were isolated as described by Mintz et al [41] with minor modifications. Briefly, nuclei were isolated from Jar or COS7.MUC1 cells at 80% confluence using the nuclear extract kit (Active Motif, Carlsbad CA). Further extraction steps were followed as described in the protocol up to the last ultracentrifugation step where the final fraction was spun at 157,000×g in a benchtop Beckman Optima TLX ultracentrifuge (TLS55 swinging bucket rotor) at 4°C for 90 min. The pellet was resuspended in LDS sample buffer and analyzed by Western blotting as described above.

### Native Gel Electrophoresis

A nuclear speckle fraction was isolated as described above. The final pellet was resuspended in TM buffer (10 mM Tris HCl pH 7.5, 5 mM MgCl2) and sonicated 3 times 5 sec each using a Branson 250 sonifier. The suspension was treated with RNase A 100 µg/ml for 2 h at room temperature. The sample was spun at high speed 2 min, the supernatant was mixed with 2X native Tris-glycine sample buffer and loaded onto a 3–8% Tris-acetate gel. The gel was run for 3 h with Tris-glycine native running buffer. The proteins were transferred onto PVDF membrane using NuPAGE transfer buffer plus 10% methanol, at constant voltage of 30 V for 20 h. The membrane was blocked, incubated with primary antibodies overnight and developed as described above.

## Results

### MUC1 Extracellular Domain Antibodies B27.29, HMFG1, and DF3 Produce an Apparent Nucleus-associated Staining in Epithelial Cells

**Figure 1:**
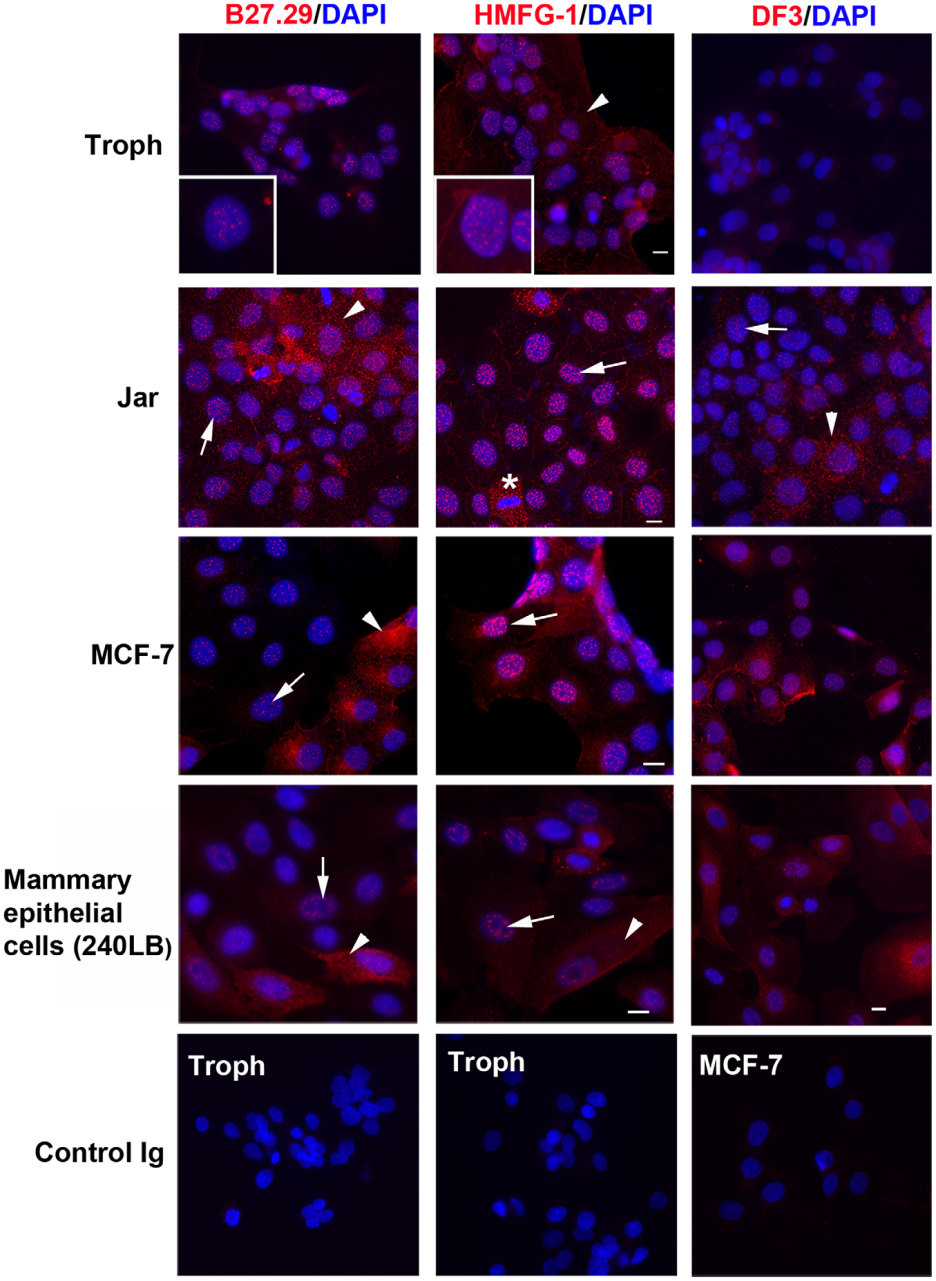
MUC1 extracellular domain antibody staining patterns in cultured epithelial cells. The indicated cells were fixed, permeabilized and stained for immunofluorescence microscopy using B27.29, HMFG1, DF3, or matched isotype control immunoglobulins (Control Ig) antibodies as described in Methods. DAPI was used to identify nuclei. The insets are higher magnification views of selected nuclei in trophoblast cells (Troph) to more clearly reveal the speckled fluorescence pattern. Arrows indicate other examples of nucleus-associated staining and arrow heads indicate examples of cytoplasmic/membrane staining. The asterisk indicates dividing cells in which HMFG1 staining is excluded from the nucleus. Images are representative of 3–8 independent experiments. The white bars represent 10 µm.

We previously noticed that antibody B27.29 produced an unexpected and apparently nucleus-associated staining pattern in rhesus monkey trophoblast cells in addition to the expected irregular punctate plasma membrane/cytoplasmic staining pattern [30]. The images in Fig. 1 confirm this (see arrows) and extend the observation to Jar cells (a human trophoblast-derived choriocarcinoma cell line), MCF-7 breast adenocarcinoma cells, and 240LB cells (normal human mammary epithelial cells). A speckled nucleus-associated staining pattern was also seen with two other widely used MUC1 extracellular domain antibodies, HMFG1 and DF3 (Fig. 1). It should be noted, however, that DF3 reacted poorly with trophoblasts. With each of the antibodies, punctate staining was also found over the plasma membrane/cytoplasm (see arrow heads in Fig. 1) for all cell types although the intensity of staining varied between cell types and even between different cells in the same culture. The speckled nucleus-associated staining was absent from nuclei in dividing cells which instead showed intense foci in the cytoplasm (see for example the Jar cells indicated by the asterisk in Fig. 1).

**Figure 2:**
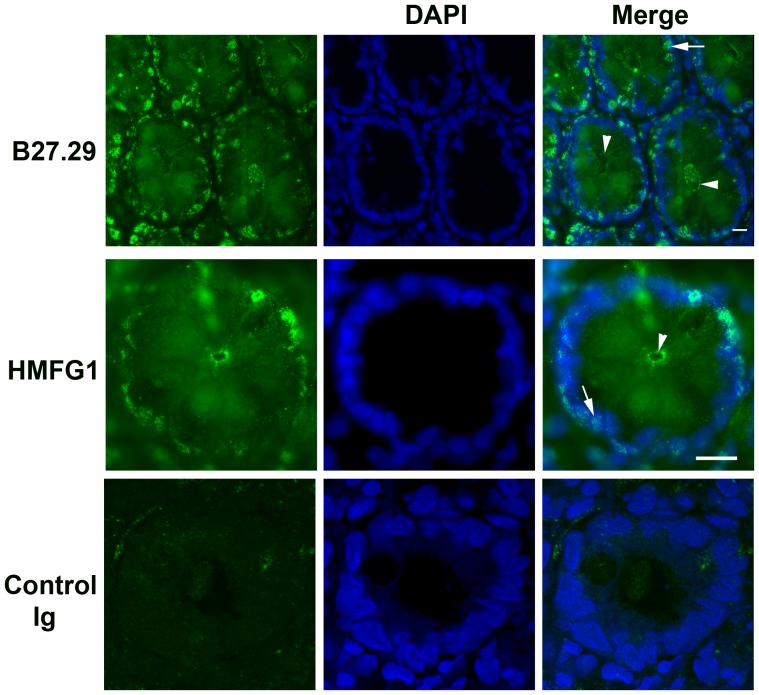
MUC1 extracellular domain antibody staining pattern in colonic tissue. Cryosections of rhesus monkey colonic tissues were processed for staining with B27.29 and HMFG1 antibodies as described in Methods. Nuclei were identified using DAPI. Arrows indicate examples of nucleus-associated staining and arrow heads indicate examples of apical plasma membrane staining of ductal epithelial cells. The white bar represents 10 µm. Images are representative of 3 independent experiments.

Next, we examined sections of rhesus monkey colon using antibodies B27.29 and HMFG1. The antibodies produced a speckled/granular nucleus-associated staining pattern in luminal epithelial cells within colonic crypts (Fig. 2, arrows). As expected, staining of the apical plasma membrane was also observed (Fig. 2, arrow heads) along with weak diffuse cytoplasmic staining.

### The B27.29- and HMFG1-reactive Proteins Localize to an Intranuclear Compartment

**Figure 3:**
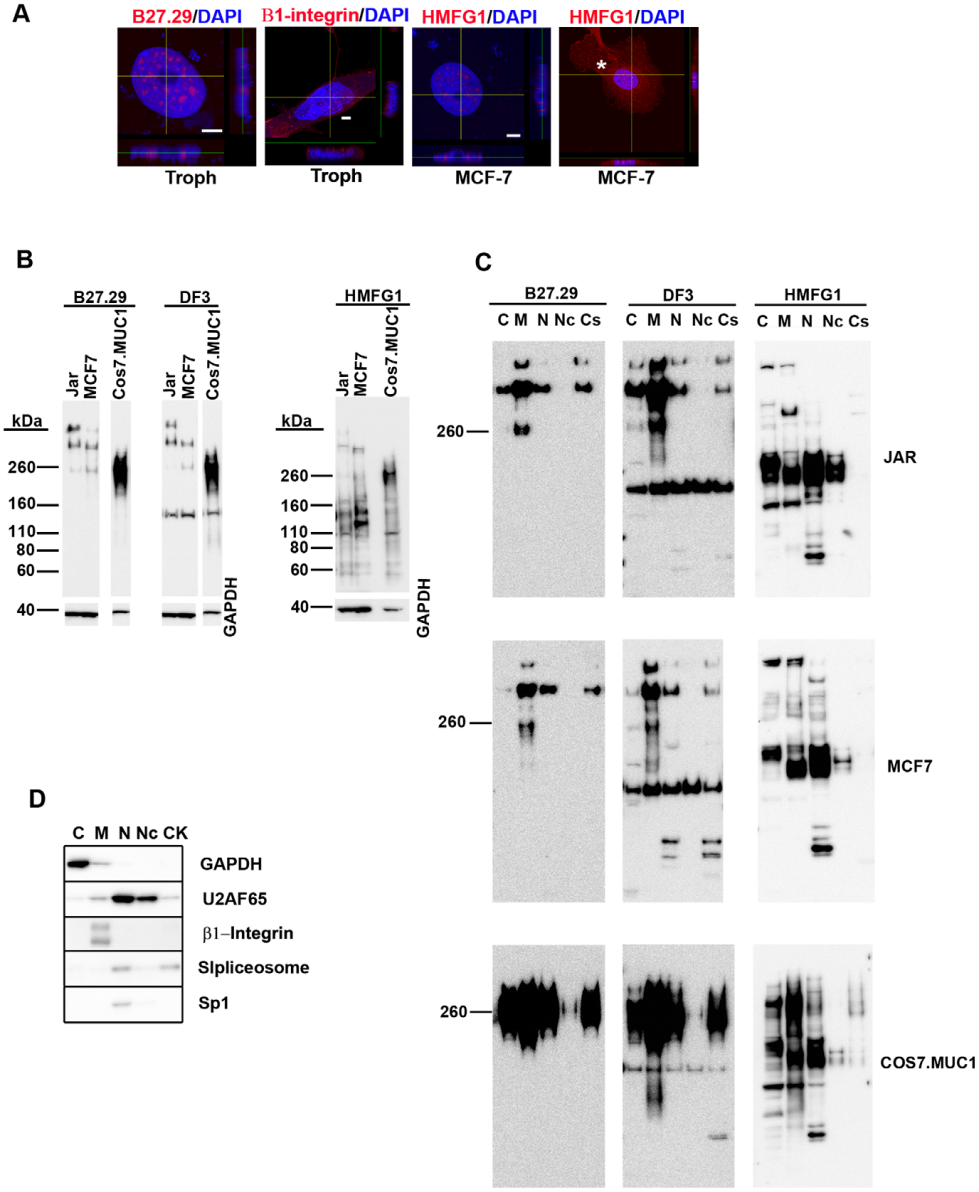
Nuclear localization of MUC1 extracellular domain antibody-reactive proteins. (A) Trophoblasts (troph) and MCF-7 cells were stained with B27.29, HMFG1, or β1-integrin antibodies (each shown in red) and then examined by confocal microscopy. Nuclei were stained using DAPI (blue). The images show the staining patterns roughly midway through the respective z-series. Lateral projections (the plane of view is indicated by the yellow horizontal and vertical lines) of individual z-stack series are shown below and to the right of each image. The bars represent 5 µm. The asterisk indicates cytoplasmic/membrane staining. (B) Western blot analysis of total lysates obtained from Jar, MCF-7, and COS7.MUC1 cells using B27.29, DF3, and HMFG1 antibodies. (C) Western blot analysis of subcellular fractions prepared from Jar, MCF-7, and COS7.MUC1 cells using MUC1 antibodies. (D) Western blot analysis of subcellular fractions using antibodies against marker proteins (GAPDH, U2AF65, β1-integrin, spliceosomes, and Sp1). The abbreviations for the subcellular fractions are, C; cytoplasmic, M; membrane, N; nuclear soluble, Nc; nuclear chromatin, Cs; cytoskeletal. The images are representative of 3–5 independent experiments.

The above results suggested an association of the B27.29- and HMFG1-reactive protein with the nucleus. To confirm this, trophoblasts and MCF-7 cells were stained with B27.29 or HMFG1 antibodies and examined by confocal microscopy. Examination of orthogonal projections of z-stack images showed that reactivity for each antibody was localized within nuclei (Fig. 3A). Specifically, fluorescent speckles were localized within DAPI^low^ regions of the nucleus but excluded the nucleolus. Irregular-shaped speckles and smaller granule-like structures, which often appeared to interconnect, were seen. For comparison, and to eliminate the possibility that the nuclear localization of the MUC1 antibody staining resulted from a fixation artifact, we also carried out confocal microscopic analysis of an unrelated plasma membrane protein, β1-integrin. In trophoblasts, β1-integrin is expressed on the apical plasma membrane [42]. Examination of confocal z-stack images showed predominant apical expression of β1-integrin and no intra-nuclear expression was found (Fig. 3A). The lower magnification confocal image (Fig. 3A, right) shows that in addition to nuclear staining, HMFG1 also produced the expected cytoplasmic/plasma membrane staining. It should be noted that MUC1 expression loses apical plasma membrane polarity in tumor cells and instead shows a broad cytoplasmic/membrane expression pattern [9], [33].

Confirmation of nuclear localization was also addressed by examination of subcellular fractions by Western blotting (Fig. 3B and 3C). For these studies we used Jar, MCF-7, and COS7.MUC1 cells. In total lysates (Fig. 3B), B27.29 and DF3 detected several bands >250 kDa; DF3 additionally detected a band of about 160 kDa. These Western blot patterns are typical of results reported for other cell types using these antibodies [43], [44], [45], [46]. HMFG1 detected several bands between 110–160 kDa. In Jar and MCF-7 cells weaker intensity bands >250 kDa were also found. A prominent 260 kDa band was found in COS7.MUC1 cells. Previous studies with other cell types have shown that HMFG1 detects a 160 kDa band and bands >200 kDa in total cell extracts [27], [47], [48] although the 160 kDa band is not always detected [49].

Analysis of subcellular fractions (Fig. 3C) using B27.29, DF3, and HMFG1 showed that while the most intense >250 kDa bands were found in the membrane fraction, these bands were also present in the cytoplasmic, nuclear soluble, and cytoskeletal fractions. No bands >250 kDa were detected in the nuclear chromatin fraction. However, the 150–160 kDa bands detected by DF3 and HMFG1 were present in all the fractions, including the nuclear chromatin fraction. The purity of the fractions was confirmed using antibodies against marker proteins (GAPDH, β1-integrin, Sp1 transcription factor, spliceosomes, and U2AF65) (Fig. 3D). These results indicate that all three MUC1 extracellular domain antibodies detect proteins >250 kDa in the nuclear soluble fraction. The nuclear soluble fraction also contained the 110–160 kDa bands detected by DF3 and HMFG1.

### The Nucleus-associated MUC1-N Antibody-reactive Protein is a *MUC1* Gene Product

**Figure 4:**
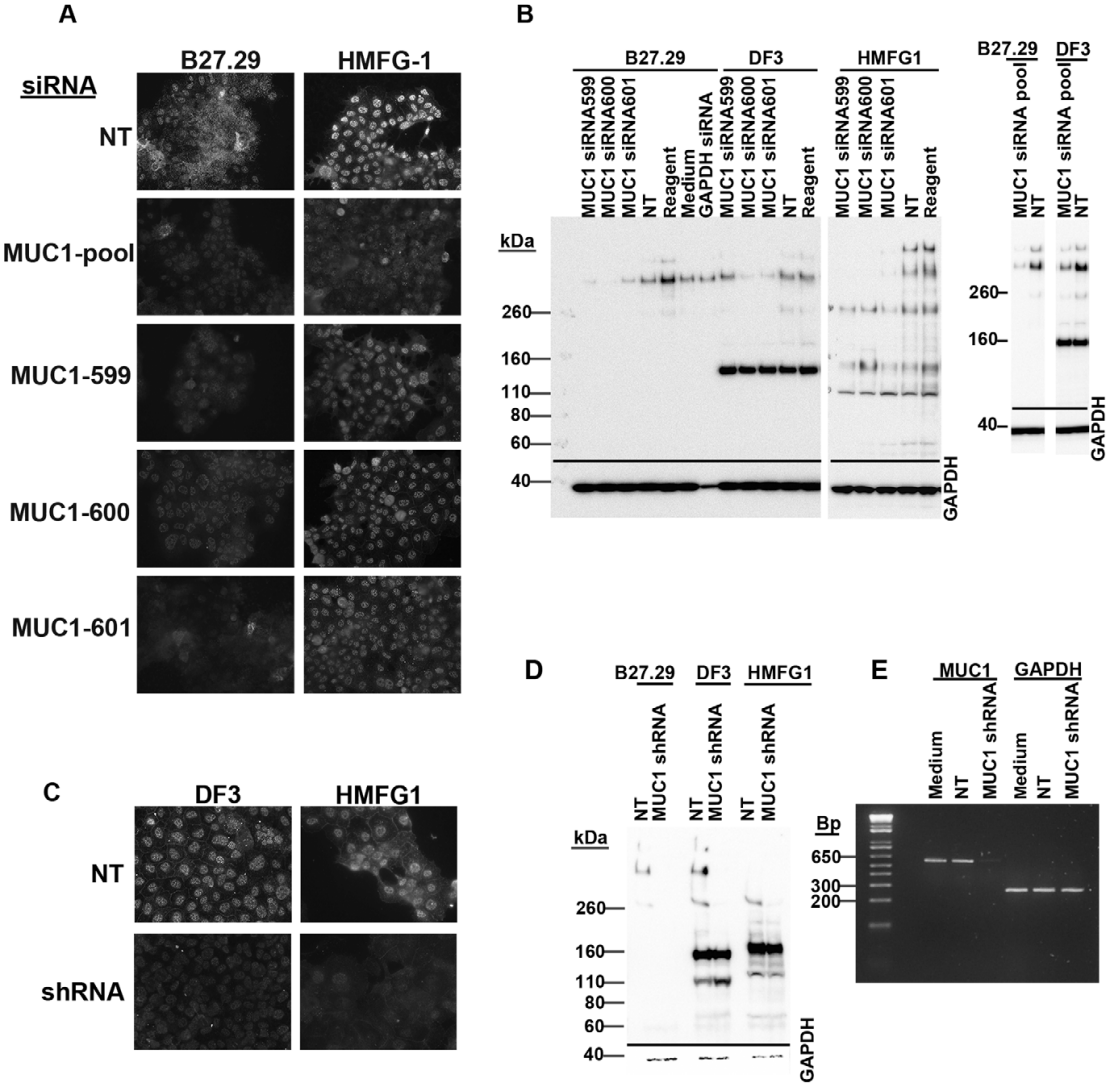
Effect of MUC1 siRNAs and shRNA on nuclear MUC1 expression. Jar cells were transfected independently with four different MUC1 siRNAs, non-targeting siRNA (NT), or GAPDH siRNA (see Methods). Five days after transfection cells were (A) stained using B27.29 or HMFG1 or (B) lysed and analyzed by Western blotting. In other experiments, Jar cells were stably transfected with MUC1 shRNA as described in Methods and stained using DF3 and HMFG1 antibodies (C) or lysed and analyzed for MUC1 expression by Western blotting (D) and RT-PCR (E). GAPDH was used as a loading control for Western blotting and RT-PCR. NT; non-targeting control. Reagent; transfection reagent alone. Medium; culture medium alone.

To confirm that the nuclear antigens recognized by MUC1 extracellular domain antibodies represent MUC1 protein and not non-specifically reacting proteins, we independently transfected Jar cells using several MUC1 siRNAs that span different regions of MUC1 mRNA. After transfection, MUC1 expression was assessed by immunofluorescence microscopy and Western blotting. The results (Fig. 4A) show that the intensity of nuclear fluorescence detected using B27.29 or HMFG1 was reduced in Jar cells transfected with each of the MUC1 siRNAs compared to cells transfected with non-targeting control siRNA. These observations along with the fact that similar results were obtained with each of the MUC1 siRNAs targeting different regions of MUC1 strongly argues that the knockdown of MUC1 expression did not result from off-target effects. It should be noted that knock-down of the nuclear MUC1 staining was not complete and was observed 5 days after transfection. If the cells were stained 2–3 days after transfection there was little or no evidence of nuclear MUC1 knock-down (results not shown).

When Western blots were probed using B27.29 or HMFG1 antibodies, the >250 kDa bands were reduced/absent in lysates from cells transfected with each of the different MUC1 siRNAs (Fig. 4B). When DF3 was used, reduced expression of the >250 kDa bands was seen for two out of the three siRNAs. Cells transfected with the non-targeting control siRNA or with GAPDH siRNA showed no loss of the >250 kDa bands detected with B27.29. Significant silencing of GAPDH expression was seen using the GAPDH siRNA but not with any of the MUC1 siRNAs. In contrast to the consistent knockdown of the >250 kDa bands, the effects of siRNA transfection on expression of the 110–160 kDa band(s) detected with HMFG1 and DF3 were inconsistent in multiple experiments; in some experiments band intensity was decreased while in others no change was observed.

Because of uncertainties regarding the identity of the 110–160 kDa bands, we carried out additional experiments using Jar cells stably transfected with MUC1 shRNA. Immunofluorescence analysis using DF3 and HMFG1 showed reduced nuclear speckle intensity and absence of cytoplasmic/membrane staining (Fig. 4C) in cells transfected with the MUC1 shRNA compared to cells transfected with NT shRNA. Western blot analysis consistently showed loss of the >250 kDa bands detected by B27.29, HMFG1, and DF3 in the MUC1 shRNA-transfected cells (Fig. 4D). However, the 110–160 kDa bands detected by HMFG1 and DF3 were unaffected by transfection with MUC1 shRNA. RT-PCR analysis confirmed knockdown of MUC1 mRNA in cells transfected with MUC1 shRNA (Fig. 4E).

Together, these results are consistent with the idea that most of the nuclear staining detected using the MUC1 extracellular domain antibodies represents a *MUC1* gene product >250 kDa. However, the results also indicate that the 110–160 kDa bands detected by HMFG1 and DF3 are likely non-specifically reacting proteins and could perhaps explain the inability to completely eliminate MUC1 nuclear speckles following siRNA/shRNA treatment. The observation that no 110–160 kDa bands were detected with B27.29 yet the antibody produced a speckled nuclear staining pattern that was reduced by siRNA or shRNA treatment further supports the idea that nuclear MUC1 detected by immunofluorescence represents the >250 kDa protein and not the 110–160 kDa species. These findings also indicate that the MUC1 antibodies react differently when used for Western blotting and immunocytochemistry and that care should be taken in the interpretation of results.

### The MUC1 Extracellular Domain Associates with Spliceosome Components in Nuclear Speckles

**Figure 5:**
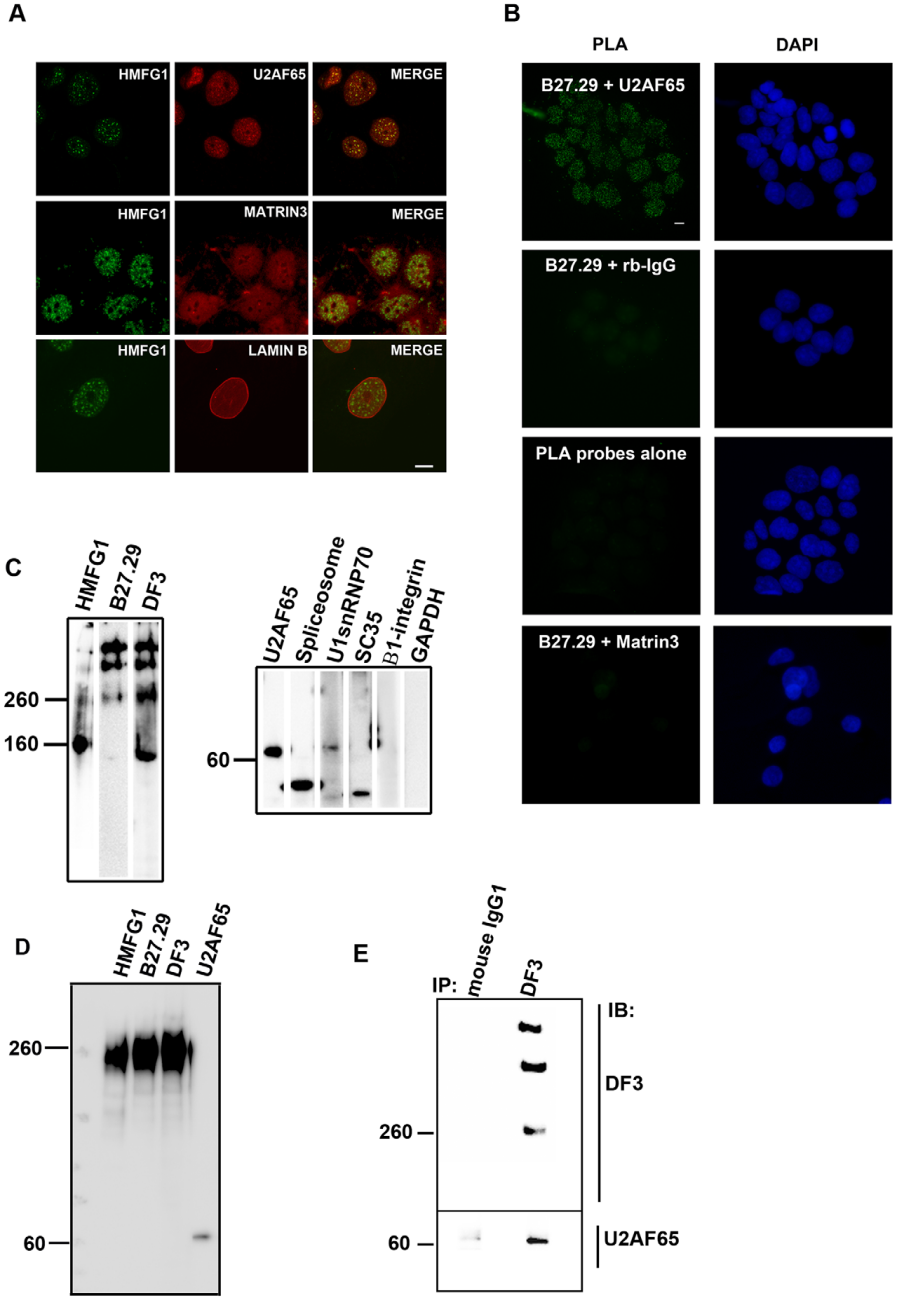
Nuclear MUC1 localizes with the spliceosome protein, U2AF65. (A) Trophoblasts were double-stained stained with HMFG1 (Green) and either antibody against U2AF65, matrin-3, or lamin B1 (all shown in Red). The cells were then examined by confocal microscopy. Representative images from the middle of the respective z-stacks are shown. Where there is overlap of green and red in the merged images, a yellow color is produced. (B) Proximity Ligation Assay of MUC1 and U2AF65**.** BeWo choriocarcinoma cells were fixed, permeabilized and processed for the proximity ligation assay (PLA) using the indicated combinations of primary antibodies/control immunoglobulin as described in Methods. Illustrated controls consist of non-immune rabbit IgG (rb-IgG; used in place of U2AF65), PLA probes alone (no primary antibodies), and B27.29 plus the nuclear matrix protein, matrin-3. Negative results were also obtained using control mouse Ig (in place of B27.29) and U2AF65 (not shown). This experiment was carried out three times with identical results. The white horizontal bars on the micrographs represent 5 µm. (C) Nuclear speckles were isolated from Jar cells and analyzed by Western blotting using HMFG1, B27.29, or DF3 antibodies. The blot on the right of C shows analysis of the nuclear speckle fraction using antibodies against marker proteins. (D) Nuclear speckles were isolated from COS7.MUC1 cells and analyzed by Western blotting. (E) Jar cells were lysed and analyzed by immunoprecipitation (IP) using antibody DF3 or control mouse Ig as described in Methods. Immunoprecipitates were subjected to Western blotting (IB) and probed using DF3 antibody or antibody against U2AF65, as indicated. The numbers represent molecular mass (kDa).

The speckled appearance of the nuclear MUC1 staining resembled the characteristic pattern seen after immunocytochemical localization of interchromatin granule clusters (“nuclear speckles”) [40], [50]. To identify the intranuclear compartment in which the MUC1 extracellular domain was located, we double-stained trophoblasts with HMFG1 and either an antibody against the non-snRNP spliceosome protein U2AF65 (a marker for nuclear speckles [51], [52]), an antibody against the nuclear matrix protein, matrin-3 [53], or an antibody against the nuclear lamina protein, lamin B1. When examined by confocal microscopy (Fig. 5A), staining with the anti-U2AF65 antibody produced a characteristic speckled nuclear fluorescence with some lower intensity nucleoplasmic staining while the matrin-3 antibody produced a characteristic diffuse, slightly granular nuclear fluorescence and some cytoplasmic staining. Lamin B1 was detected as bright fluorescence at the nuclear envelope and weaker diffuse fluorescence throughout the nucleoplasm. Examination of the double-stained samples (Fig. 5A) suggests that the HMFG1-reactive antigen colocalizes with U2AF65 in nuclear speckles (yellow speckles in the merged image) but not with matrin-3 or lamin B (green speckles in the merged images).

To further substantiate the association between the MUC1 extracellular domain and U2AF65, we used an in situ proximity ligation assay (PLA) which allows immunocytochemical visualization, localization, and quantification of protein-protein interactions [54], [55], [56], [57], [58], [59]. BeWo cells were first incubated with both B27.29 and anti-U2AF65 antibodies. Secondary antibodies labeled with unique short DNA strands (PLA probes) were then added. If the bound probes are in close proximity they can serve to generate circular DNA strands which in turn serve as a template for a rolling circle amplification reaction. The amplification product is then detected using fluorescently labeled complementary oligonucleotides. The maximum distance between epitopes required for the formation of amplifiable ligation products is estimated to be about 30–40 nm, based on known antibody and oligonucleotide sizes [57] (and Olink, personal communication).

The results (Fig. 5B) of the PLA show abundant small bright fluorescent foci in the nuclei of cells double-stained using B27.29 and anti-U2AF65. No staining was detected in the cytoplasm. When the PLA was carried out using cells double-stained with B27.29 and control rabbit IgG (the control for the anti-U2AF65 antibody), very little or no fluorescence was detected. Cells incubated with PLA probes alone also failed to show any fluorescence. The PLA was also used to test for interaction between the MUC1 extracellular domain and the nuclear matrix protein, matrin-3. The results showed the absence of nuclear fluorescence. These results suggest that the MUC1 VNTR epitope detected using B27.29 and an epitope(s) on U2AF65 are in close proximity with about 40 nm maximum separation.

Next, we isolated nuclear speckles from Jar cells and carried out a Western blot analysis using MUC1 antibodies. The results in Fig. 5C show that the >250 kDa bands as well as the 160 kDa band were found in the nuclear speckle fraction using HMFG1, B27.29, and DF3 antibodies. The purity of the speckle fraction was assessed by staining the blots with antibodies against various marker proteins. Bands were detected using antibodies against U2AF65, spliceosomes, U1snRNP70, and SC35 but no band was detected using antibodies against β1-integrin (plasma membrane marker) or GAPDH (cytoplasmic marker). Speckles were also isolated from COS7.MUC1 cells and Western blot analysis (Fig. 5D) showed the presence of a strong 260 kDa band using all three MUC1 extracellular domain antibodies. The same fraction also contained U2AF65.

Finally, we tested for an association between MUC1 and U2AF65 using immunoprecipitation analysis. When Jar cell nuclear extracts were incubated with anti-MUC1 antibody (DF3) the resulting immunoprecipitates were found to contain U2AF65 as well as MUC1 (Fig. 5E). Little or no U2AF65 or MUC1 was detected when immunoprecipitation was carried out using control mouse Ig.

Together these results indicate that MUC1-N associates with the spliceosome protein U2AF65.

### Nuclear Localization of the MUC1 Extracellular Domain Requires RNA

**Figure 6:**
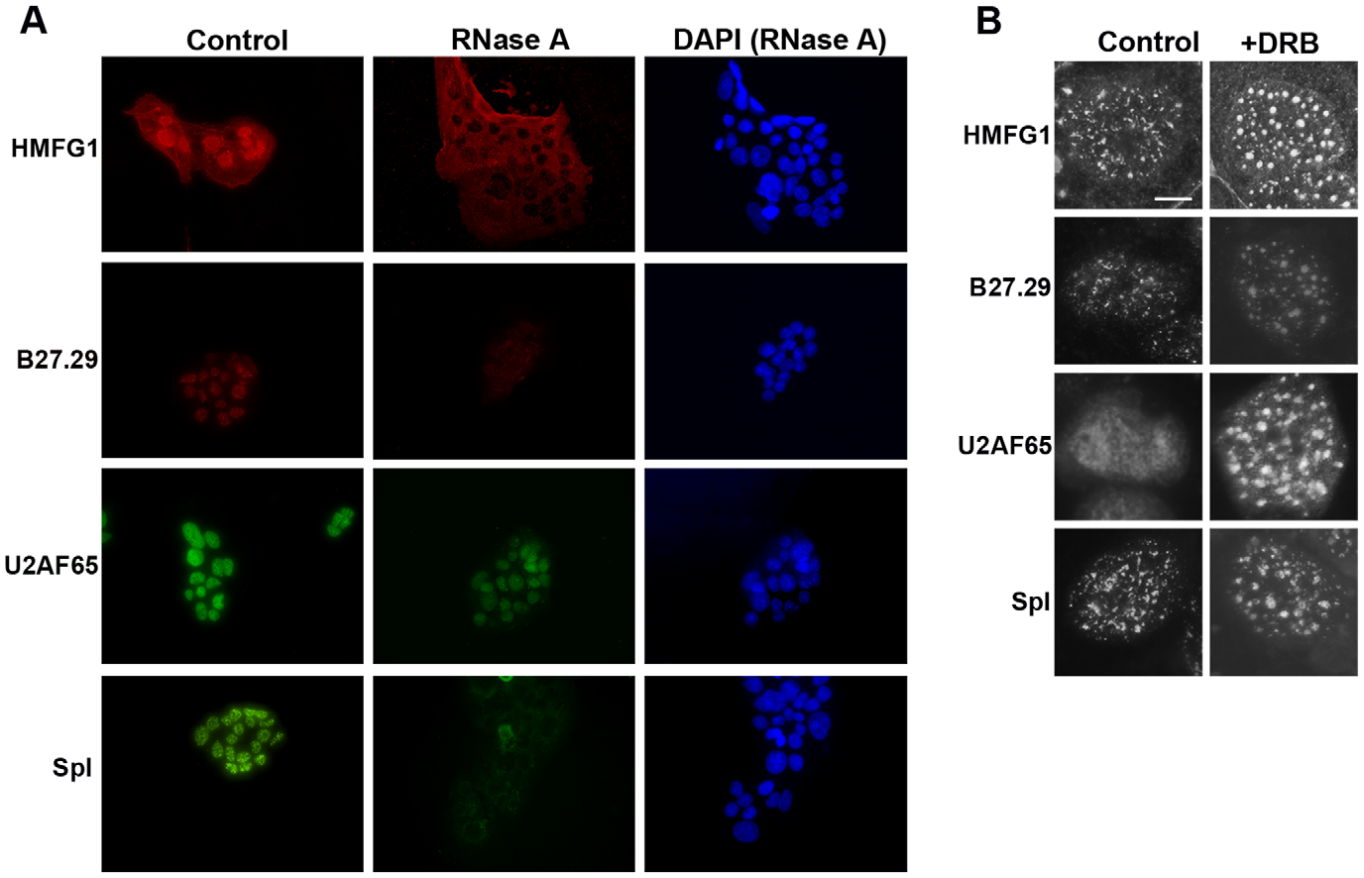
Effect of RNase A and transcriptional inhibition on the intranuclear distribution of MUC1. (A) BeWo cells were fixed and permeabilized using methanol and then incubated with RNase A (100 µg/mL) for 2 h as described in Methods. The cells were then stained with antibodies against MUC1 (HMFG1 and B27.29), U2AF65, and spliceosomes as indicated. Nuclei were stained with DAPI. (B) BeWo cells were incubated in the presence of DRB (100 µM) for 2 h and then stained with antibodies against MUC1, U2AF65, and spliceosomes (Spl) as described in Methods. The bar represents 5 µm. Results are representative of 3 independent experiments.

The organization of some spliceosomal proteins in nuclear speckles requires an interaction with RNA. We therefore examined the dependency of MUC1 nuclear localization on RNA. BeWo cells were fixed and permeabilized using methanol, incubated with RNase A, and then stained for MUC1. Cells were also stained for U2AF65 and spliceosomal snRNPs. It can be seen from Fig. 6A that nuclear localization of MUC1 was lost after RNase A treatment and the nuclear staining intensity of U2AF65 was reduced. The speckled nuclear expression of snRNPs was also lost after RNase treatment, as described by others [40], [60].

Next, we tested whether the nuclear localization of MUC1 was dependent on spliceosome activity. For this, cells were incubated with 5,6-dichloro-1-*b*-d-ribofuranosylbenzimidazole (DRB), an inhibitor of RNA polymerase II [61]. Compared to untreated controls, HMFG1- and B27.29-positive nuclear speckles in DRB-treated cells were larger and rounder with fewer interconnections (Fig. 6B). Similar results were found for U2AF65 and spliceosomal proteins, consistent with previous reports for the effects of transcriptional inhibition on splicing factor expression [52], [60], [62].

### Is Nuclear MUC1-N Associated with MUC1-C?

**Figure 7:**
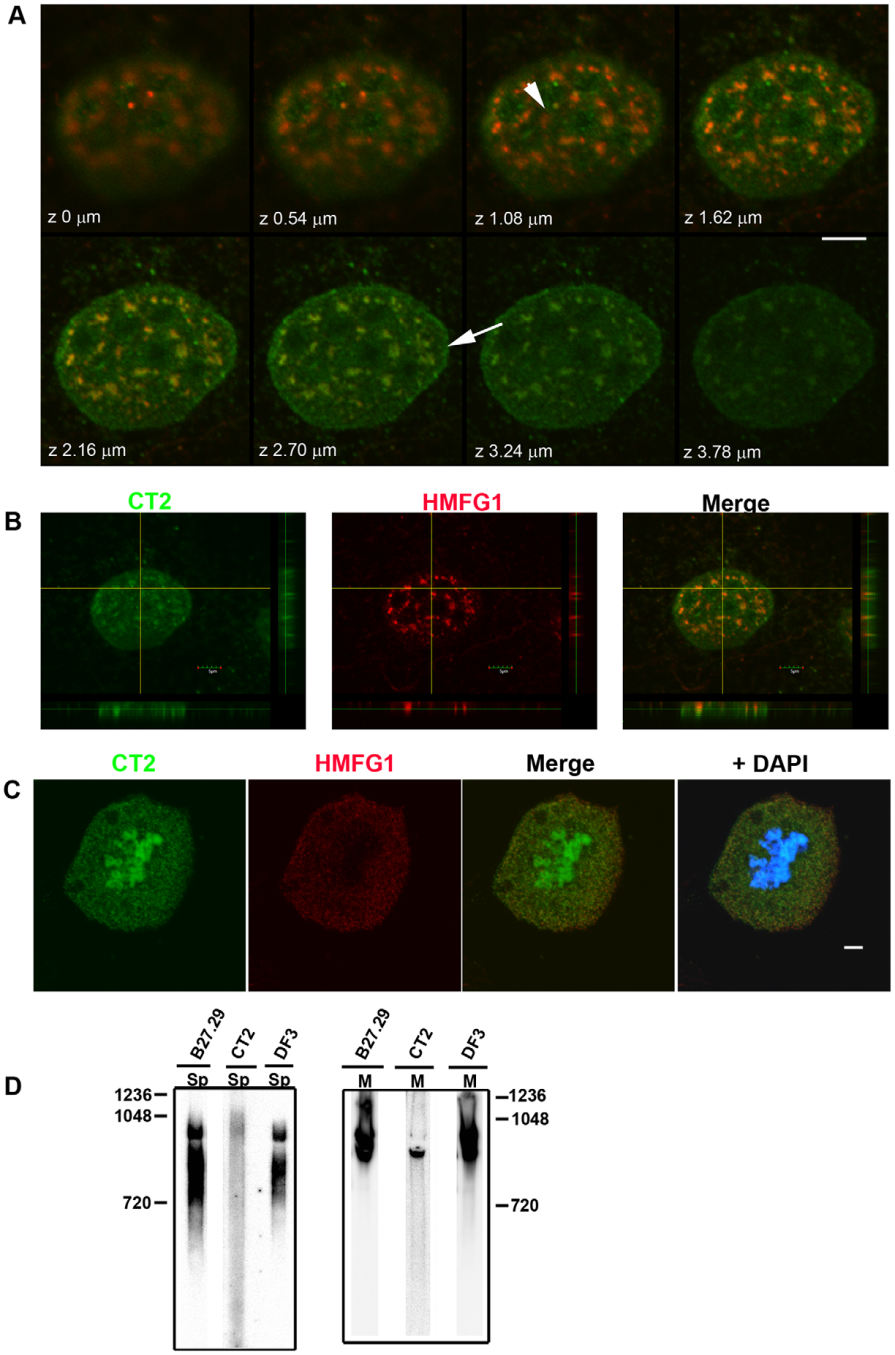
Comparison of the expression of MUC1-N and MUC1-C. (A) Confocal z-series images of Jar cell nuclei double-stained with antibodies HMFG1 (red) and the CT2 (green). The arrowhead indicates CT2 staining in a nucleolus and the arrow indicates CT2 staining at the nuclear periphery. Note the absence of HMFG1 staining in these locations. (B) Lateral projections along x and y from the same z-series shown in B. The yellow lines indicate the planes of view for the lateral projections. Note the more apical distribution of HMFG1 staining and the more basal staining of CT2 staining. (C) Single section confocal images of a Jar cell in metaphase and stained with HMFG1 and CT2. The white horizontal bars indicate 5 µm. (D) Nuclear speckles (Sp) were isolated from Jar cells and analyzed by native polyacrylamide gel electrophoresis followed by Western blotting using the indicated antibodies. The same antibodies were used to analyze Western blots prepared from Jar membrane (M) fractions. Note the prominent band detected by CT2 in the membrane fraction was not found in nuclear speckles. The numbers represent molecular mass (kDa). Results are representative of 3 independent experiments.

Figure 7A shows confocal images of Jar cell nuclei stained with HMFG1 and CT2. CT2 produced a diffuse granular fluorescence pattern in most nuclei. In some, but not all, nuclei larger fluorescent foci were evident. CT2 also stained nucleolar regions and areas at the nuclear periphery. Examination of merged CT2 and HMFG1 staining patterns confirmed that the diffuse CT2 staining within the nuclear matrix was not associated with HMFG1 staining. HMFG1 also differed from CT2 in that it did not produce nucleolar staining or staining of the nuclear periphery. It was more difficult to assess the colocalization of CT2 and HMFG1 reactivity in speckled structures due to the diffuse background CT2 staining. Examination of sequential z-stack images (Fig. 7A) and lateral projections of the z-series (Fig. 7B) showed that while some speckled structures appeared to express both CT2 and HMFG1, the fluorescence signals were separated in the z-dimension with only partial overlap; CT2 staining in speckled structures occurred predominantly in the more basal optical sections whereas HMFG1-reactive speckled structures were predominantly found in the more apical sections of nuclei. Some HMFG1-positive speckles were not associated with CT2-positive speckled structures. Additional evidence for differential localization of MUC1-N and MUC1-C within nuclei was obtained from examination of mitotic cells. Figure 7C shows a Jar cell at metaphase stained for HMFG1, CT2 and DAPI and it can be seen that there was little or no co-localization of the two fluorescence signals. Attempts to detect interaction between MUC1-N and MUC1-C using the proximity ligation assay were not useful; while a weak nuclear fluorescence was detected using B27.29 and CT2, a similar result was obtained using B27.29 and Armenian hamster IgG (control for CT2; Results not shown).

Next, we looked to see whether the CT2 antibody could detect protein in the isolated nuclear speckle fraction. For this, purified nuclear speckles from Jar cells were subjected to native polyacrylamide gel electrophoresis and then analyzed by Western blotting. Although we detected high molecular mass bands using B27.29 and DF3, no band was detected using CT2. However, all three antibodies detected bands on native Western blots prepared from the membrane fraction of Jar cells (Fig. 7D). These results are consistent with the idea that MUC1-N but not MUC1-C is present in nuclear speckles.

## Discussion

The results presented here show for the first time that the extracellular domain subunit of the mucin, MUC1, is expressed in interchromatin granule clusters (nuclear speckles) within nuclei of trophoblasts and other normal and cancerous epithelial cells. These conclusions are supported by comprehensive experimental data obtained using three different antibodies against the extracellular domain of MUC1, four different MUC1 siRNAs, and MUC1 shRNA. Previous immunocytochemical and immunoprecipitation studies have detected the MUC1-C subunit (which comprises the cytoplasmic domain, the transmembrane domain, and a short extracellular domain that does not include the VNTR region) in the nucleus of breast cancer cell lines and breast cancer tissue but failed to detect MUC1 extracellular domain (i.e., the N-terminal subunit) in that location. These findings have supported the widely held view that only MUC1-C translocates to the nucleus possibly after autoproteolysis and/or after shedding of the extracellular domain [13], [16], [18], [22]. However, in addition to the new data reported here, there have been other observations that challenge this idea although they have not received widespread acknowledgment in the literature. MUC1 extracellular domain is found in the cytoplasm of tumor cells and some normal epithelial cells and a Western blotting study suggested the presence of MUC1-N in a nuclear fraction derived from breast cancer tissue [11]. Francis et al [63] noted that MUC1 extracellular domain antibody reactivity was associated with the nucleus in progesterone-stimulated endometrial epithelial cells but did not pursue or discuss the observation further. Cascio et al [28] recently reported that the extracellular domain of MUC1 forms a complex with p65 and that the complex associates with cytokine promoter regions in the nuclei of breast cancer cells.

Our findings must be considered in the light of the many immunohistochemical and immunocytochemical studies using MUC1 extracellular domain antibodies that have failed to describe any nuclear localization. Sometimes failure to report nuclear localization in tissue sections is due to low magnification images which preclude adequate assessment of nuclear staining. However, in some cases nuclear staining is evident from photomicrographs but is not mentioned or discussed by the authors (e.g., [64], [65]). In a survey of fifty-six MUC1 antibodies, Cao et al [66] reported that some antibodies showed a Golgi localization pattern when used to stain T-47D breast carcinoma cells. Unfortunately, the image quality makes it difficult to assess this conclusion and to distinguish Golgi from nuclear staining. Some of the studies that fail to show nuclear localization of MUC1-N used the same antibodies as used here [67], [68], [69]. In some cases, failure to detect nuclear staining is due to lack of cell permeabilization (e.g., [69]). Other reasons for negative conclusions regarding the nuclear expression of MUC1-N include the variability of MUC1 extracellular domain antibodies with regards the effects of glycosylation on epitope recognition and the variability in the extent of glycosylation of MUC1 in different cells and tissues. For example, while we found intense nuclear staining in all cells studied using B27.29 and HMFG1 antibodies, another MUC1 extracellular domain antibody, DF3, produced weaker nuclear staining in MCF7 and BeWo cells and no nuclear staining in trophoblasts. Wang et al [27] reported that HMFG1 produced intracellular (but not nuclear) staining in human uterine epithelial cells whereas another MUC1 antibody (214D4) produced only cell surface staining.

While we consistently found nuclear localization of MUC1-N in all cell types and tissues studied, some cells (e.g., MCF-7) showed a heterogeneous staining pattern in which antibody reactivity was found predominantly in the nucleus of some cells while others had a predominantly plasma membrane/cytoplasmic expression pattern with no evidence of nuclear localization. A similar heterogeneous expression pattern was found for primary cultures of normal human mammary epithelial cells indicating that this distribution is not unique to cancer cells. The absence of any change is this pattern when cells were treated with mitomycin C makes it unlikely that the differential MUC1 expression in MCF-7 cells is cell cycle stage-dependent (results not shown). It is possible that the nuclear MUC1-expressing cells in the MCF-7 cultures represent a distinct subpopulation. This idea is supported by a previous report of “side populations” with stem cell characteristics within the MCF-7 cell line [70]. Interestingly, in this same study, 77% of the MCF-7 side population expressed MUC1 on the cell surface whereas the remaining side population cells only expressed “intracellular” MUC1. While these experiments used flow cytometry and the precise intracellular compartment was not defined, it is possible that the nuclear MUC1 expressing cells found in MCF-7 cultures in the present study correspond to this subpopulation of cells described by Engelman et al [70]. The significance of these observations remains to be determined.

The siRNA and shRNA studies confirm that the nuclear reactivity detected by B27.29, HMFG1, and DF3 antibodies represents a *MUC1* gene product. The specificity of HMFG1 for the VNTR region of MUC-N has been demonstrated in other studies [49], [71], [72]. The specificity of B27.29 for the VNTR region has also been established [4], [73]. While the nuclear MUC1 staining could be reduced using MUC1 siRNAs this effect was not observed in Jar cells until several days after transfection suggesting that the nuclear MUC1-N has a relatively long half-life. It should be noted that while the higher molecular mass MUC1 bands (>250 kDa) were consistently knocked down by siRNA or shRNA treatment, knockdown of the lower molecular mass band(s) (110–160 kDa) was inconsistent (siRNA) or not observed (shRNA). The exact nature of the 110–160 kDa bands therefore remains to be determined but the results obtained here suggest that they likely represent non-specific antibody binding and that caution should be used in the interpretation of Western blots using HMFG1 and DF3. These findings also demonstrate differences in the reactivity of the MUC1 antibodies depending on whether they are used for Western blot or immunocytochemistry. Since we observed reduced immunofluorescence staining (both membrane/cytoplasmic and nuclear) when cells were treated with MUC1 siRNAs or shRNA and consistent knockdown of the >250 kDa bands by Western blot, we conclude that nuclear MUC1-N is represented by the latter species and not the 110–160 kDa species. This conclusion is supported by the observation that while the B27.29 antibody produces speckled nuclear staining, unlike HMFG1 and DF3 it only detects >250 kDa protein bands by Western blot. However, we cannot entirely exclude the possibility that some of the nuclear immunofluorescence staining detected using HMFG1 or DF3 represents the non-specific 110–160 kDa species.

The presence of the MUC1 extracellular domain in nuclear speckles as determined by immunofluorescence, subcellular fractionation, and immunoprecipitation analyses, and the dependence of this localization on RNA suggest a possible role in the assembly and/or organization of these structures. Speckles are comprised of spliceosome components which in turn comprise large megadalton complexes consisting of hundreds of snRNPs and non-snRNPs [62], [74]. It is now thought that speckles represent sites of assembly or storage of spliceosomes and that transcription occurs on perichromatin fibrils which can be closely associated with speckles [75]. Spliceosomes catalyze the processing of pre-mRNA and are responsible for normal and alternative splicing. Spliceosome proteins are highly dynamic and shuttle from nuceloplasm to speckles (and to the cytoplasm) [62]. Our proximity ligation and immunoprecipitation results suggest that the MUC1-N subunit is closely associated with the spliceosome protein U2AF65 and that the association depends on RNA. U2AF65 is a non-snRNP that is an auxiliary factor supporting SF1 and SF3b155 (a U2 snRNP subunit) function during pre-mRNA splicing [76], [77], [78]. Interestingly, MUC1 behaved like typical snRNPs rather than non-snRNPs after treatment with the transcription inhibitor, DRB. Although the MUC1 VNTR region is rich in serine and threonine, motif analysis of the MUC1-N primary sequence failed to detect a consensus RS domain, which is known to provide a signal that targets other spliceosomal proteins to speckles [79], [80]. It is therefore unclear at this time whether MUC1-N associates with speckles via another targeting motif or whether it interacts with another protein that expresses an RS domain.

While we found both MUC1-N and MUC1-C in the nucleus, we did not find compelling evidence that they were present as a heterodimer; in contrast to MUC1-N which was localized mainly to speckles, MUC1-C was present in the nuclear matrix, nucleoli, and at the nuclear periphery. Other studies have also shown MUC1-C to be expressed in nucleoli or to have a diffuse granular expression pattern in nuclei [15], [16]. The absence of MUC1 heterodimer in the nucleus is consistent with previous immunoprecipitation studies which failed to detect the MUC1 extracellular domain subunit associated with MUC1-C in the nucleus of various cancer cell lines [13], [18]. On the other hand, Cascio et al [28] found (using chromatin immunoprecipitation) MUC1 extracellular domain and cytoplasmic domain bound to cytokine promoters in breast cancer cells stimulated with TNF-α and concluded that full length MUC1 was present in the nucleus. In the present studies, MUC1-C was found in speckle-like structures within Jar cell nuclei which in some, but not all, cases also expressed MUC1-N. However, even in these structures confocal microscopic analysis showed apparent separation of antibody reactivities with only partial overlap. Clarification of this intriguing staining pattern will require immunoelectron microscopic analysis. Additional evidence that MUC1-N and MUC1-C are localized independently in the nucleus was provided by the absence of MUC1-C in isolated nuclear speckles and by the different distribution of HMFG1 and CT2 staining in mitotic cells.

The different results regarding the nuclear expression and intranuclear localization of MUC1 may reflect differences in cell types, differences in MUC1 glycosylation, and the specificities of the different antibodies employed. The requirement for an exogenous activator to induce nuclear MUC1-C localization in other studies also differs from the results presented here. We consistently found MUC1-N in the nucleus under standard culture conditions for all cell types and nuclear localization was maintained when cells were cultured under serum-free conditions (results not shown).

Other questions which remain to be answered are how does MUC1-N enter the nucleus and what is the trafficking pathway? With regards nuclear entry, molecular size precludes passive diffusion and sequence analysis of MUC1-N using different motif analysis tools did not reveal any known nuclear localization signal. One possibility is that MUC1-N enters the nucleus associated with another protein. Examples of such a “piggy-back” mechanism are known. For example, Cdk2 requires cyclin E for nuclear import [81] and nuclear import of ElF4E requires interaction with 4E-T [82].

The trafficking pathway by which MUC1-N reaches the nucleus could involve direct transport after synthesis and autoproteolysis or could occur after the protein has been inserted in the plasma membrane (i.e. via an endocytic pathway). However, the latter pathway would require separation/cleavage of the MUC1 heterodimer at some point. Cleavage at the cell surface would result in shedding of the extracellular domain, making it difficult to see how it could reach the nucleus unless it was subsequently endocytosed. However, several secreted proteins (such as FGF) are known to be endocytosed and then translocated to the nucleus [19] and cell surface MUC1 is known to be recycled via an endocytic pathway [83], [84]. Alternatively, it is possible the intact MUC1 heterodimer is internalized by endocytosis and that cleavage occurs in some endosomal or post-endosomal compartment. It is also conceivable that MUC1 could be cleaved in the nucleus since both proteasomes and matrix-metalloproteinases (MMPs) are known to be present and function in the nucleoplasm [85], [86]. Oppizzi et al showed that full length MUC1 and another membrane-tethered mucin, β-dystroglycan, were translocated to the nucleus if SEA domain cleavage was blocked but that only the cytoplasmic domains of these proteins reached the nucleus when autoproteolysis occurred [22].

Yet another possibility is that the nuclear MUC1 species detected in the present studies represents a splice variant that lacks the cytoplasmic tail such that no cleavage would be required. The splice variant MUC1/sec fits this description [87]. Furthermore, MUC1/sec is secreted and can act as a ligand for another splice variant termed MUC1/Y which lacks the extracellular tandem repeat mucin domain [88].

An increasing number of plasma membrane proteins have been found to be translocated to the nucleus where they have diverse functions including regulation of transcription and cell proliferation [19], [21]. In the case of Notch [89] or β-dystroglycan [22] it is the cytoplasmic domain that enters the nucleus after cleavage from the extracellular domain at the plasma membrane. Nuclear translocation of MUC1-C [13] may belong to this category. In other cases, as with the fibroblast growth factor receptor (FGFR1) [90] and the epidermal growth factor receptor [91], the full length protein translocates to the nucleus. Further studies are clearly required to substantiate these speculations and to understand the role of MUC1-N in the nucleus.
