# Correction: The MUC1 Extracellular Domain Subunit Is Found in Nuclear Speckles and Associates with Spliceosomes

**DOI:** 10.1371/annotation/bb4082f7-5f88-4d64-8cab-f2e9c89b86eb

**Published:** 2012-10-26

**Authors:** Priyadarsini Kumar, Louise Lindberg, Twanda L. Thirkill, Jennifer W. Ji, Lindsay Martsching, Gordon C. Douglas

The first author's name is spelled incorrectly. The correct name is: Priyadarsini Kumar. 

